# Viability Study of Machine Learning-Based Prediction of COVID-19 Pandemic Impact in Obsessive-Compulsive Disorder Patients

**DOI:** 10.3389/fninf.2022.807584

**Published:** 2022-02-10

**Authors:** María Tubío-Fungueiriño, Eva Cernadas, Óscar F. Gonçalves, Cinto Segalas, Sara Bertolín, Lorea Mar-Barrutia, Eva Real, Manuel Fernández-Delgado, Jose M. Menchón, Sandra Carvalho, Pino Alonso, Angel Carracedo, Montse Fernández-Prieto

**Affiliations:** ^1^Genomics and Bioinformatics Group, Center for Research in Molecular Medicine and Chronic Diseases (CiMUS), Universidade de Santiago de Compostela (USC), Santiago de Compostela, Spain; ^2^Fundación Instituto de Investigación Sanitaria de Santiago de Compostela (FIDIS), Santiago de Compostela, Spain; ^3^Grupo de Medicina Xenómica, Universidade de Santiago de Compostela (USC), Santiago de Compostela, Spain; ^4^Centro Singular de Investigación en Tecnoloxías Intelixentes (CiTIUS), Universidade de Santiago de Compostela (USC), Santiago de Compostela, Spain; ^5^Proaction Lab, Faculty of Psychology and Educational Sciences, University of Coimbra, Coimbra, Portugal; ^6^Department of Physical Medicine and Rehabilitation, Spaulding Neuromodulation Center, Spaulding Rehabilitation Hospital and Harvard Medical School, Boston, MA, United States; ^7^OCD Clinical and Research Unit, Psychiatry Department, Hospital Universitari de Bellvitge, Barcelona, Spain; ^8^Institut d’ Investigació Biomèdica de Bellvitge (IDIBELL), L’Hospitalet de Llobregat, Barcelona, Spain; ^9^Department of Clinical Sciences, University of Barcelona, Barcelona, Spain; ^10^CIBERSAM (Centro de Investigación en Red de Salud Mental), Instituto de Salud Carlos III, Madrid, Spain; ^11^Translational Neuropsychology Lab, Department of Education and Psychology and William James Center for Research (WJCR), University of Aveiro, Campus Universitário de Santiago, Aveiro, Portugal; ^12^Genetics Group GC05, Instituto de Investigación Sanitaria de Santiago (IDIS), Santiago de Compostela, Spain; ^13^Grupo de Medicina Xenómica, Centro de Investigación en Red de Enfermedades Raras (CIBERER), Universidade de Santiago de Compostela (USC), Santiago de Compostela, Spain; ^14^Fundación Pública Galega de Medicina Xenómica, Servicio Galego de Saúde (SERGAS), Santiago de Compostela, Spain

**Keywords:** OCD, COVID-19, obsessive-compulsive disorder, Y-BOCS, machine learning, classification, regression

## Abstract

**Background:**

Machine learning modeling can provide valuable support in different areas of mental health, because it enables to make rapid predictions and therefore support the decision making, based on valuable data. However, few studies have applied this method to predict symptoms’ worsening, based on sociodemographic, contextual, and clinical data. Thus, we applied machine learning techniques to identify predictors of symptomatologic changes in a Spanish cohort of OCD patients during the initial phase of the COVID-19 pandemic.

**Methods:**

127 OCD patients were assessed using the Yale–Brown Obsessive-Compulsive Scale (Y-BOCS) and a structured clinical interview during the COVID-19 pandemic. Machine learning models for classification (LDA and SVM) and regression (linear regression and SVR) were constructed to predict each symptom based on patient’s sociodemographic, clinical and contextual information.

**Results:**

A Y-BOCS score prediction model was generated with 100% reliability at a score threshold of ± 6. Reliability of 100% was reached for obsessions and/or compulsions related to COVID-19. Symptoms of anxiety and depression were predicted with less reliability (correlation R of 0.58 and 0.68, respectively). The suicidal thoughts are predicted with a sensitivity of 79% and specificity of 88%. The best results are achieved by SVM and SVR.

**Conclusion:**

Our findings reveal that sociodemographic and clinical data can be used to predict changes in OCD symptomatology. Machine learning may be valuable tool for helping clinicians to rapidly identify patients at higher risk and therefore provide optimized care, especially in future pandemics. However, further validation of these models is required to ensure greater reliability of the algorithms for clinical implementation to specific objectives of interest.

## Introduction

The COVID-19 outbreak, declared on March 11, 2020 by the World Health Organization (WHO), caused an increase in symptoms of anxiety, distress and depression in the general population, due to the uncertainty of the situation. People with mental disorders experienced worsening of symptoms and, in particular, OCD patients were affected by the general measures implemented to prevent the spread of COVID-19 infections (such as hand-washing and social distancing) (Banerjee, 2020; Benatti et al., 2020; French and Lyne, 2020; Jelinek et al., 2020; Matsunaga et al., 2020).

Obsessive-Compulsive Disorder (OCD) is a psychiatric condition affecting 2–4% of the population and it is characterized by frequent, intrusive and threatening thoughts, images and ideas (obsessions) which the individual tries to control through repetitive behaviors and thoughts (compulsions) (Geller, 2006; American Psychiatric Association [APA], 2013).

Evidence suggests that people with OCD suffered a substantial impact with the current pandemic (French and Lyne, 2020; Jelinek et al., 2020; Alonso et al., 2021), especially those with contamination-related and washing-related compulsions (Fontenelle and Miguel, 2020). A recent study by Alonso et al. (2021) during the initial (and acute) stage of the lockdown in Spain showed that OCD patients experienced symptoms worsening and the appearance of COVID-19-related obsessions (e.g., fear of getting infected by SARS-CoV2). However, it was also stressed that the majority of the OCD patients (as compared to healthy controls) showed adaptive coping strategies with the emotional distress caused by the lockdown. This impact may have been moderated by several socio-emotional variables, such as resilience strategies involving online communication with relatives, physical self-care and adequate financial state (Alonso et al., 2021). It is therefore important to identify those patients at higher risk of worsening symptomatology during the pandemic in order to improve the support provided for such patients in a similar situation in the future.

The risk of worsening symptomatology can be predicted by using machine learning models, which have proved valuable in the field of psychiatry (Bzdok and Meyer-Lindenberg, 2018; Dwyer et al., 2018; Janssen et al., 2018; Xu et al., 2019; Eslami et al., 2021) for predicting the appearance of symptoms and changes in the prognosis of some disorders such as depression, anxiety and psychosis, with the aim of providing appropriate psychological and/or pharmacological treatments. Machine learning has also proved useful for predicting changes in symptomatology in OCD patients over time (Hoexter et al., 2013; Agne et al., 2020) and for predicting the severity of OCD symptoms in combination with other methods (Hoexter et al., 2013). The algorithms used in machine learning can identify and rank the most important variables for predicting a specific outcome of interest, and they are particularly useful for guiding the design of clinical studies.

For several years, the world has been alerted to the possibility of the occurrence of global pandemics. Since 2014, when the Ebola epidemic emerged in West Africa, there has been talk of the possibility of a SARS pandemic that would entail devastating economic losses worldwide (Castillo-Chavez et al., 2015). Scientists continue to maintain this discourse, warning that other pandemics, like that caused by COVID-19, will occur (Dodds, 2019; Howard et al., 2020; Mason and Friese, 2020).

It is important have tools to predict how the severity of psychiatric patients’ symptoms might change with the onset of a new pandemic.

The main objective of this study was to predict clinical changes in a cohort of OCD patients during the initial stages of the COVID-19 pandemic, based on contextual, sociodemographic and clinical variables, by using advanced machine learning models. Being able to predict worsening symptoms in conditions similar to the COVID-19 pandemic will guide the development of more effective treatments for such patients in future pandemics.

## Materials and Methods

### Participants

A total of 127 adults with OCD with ages between 18 and 65 years old (68 females; mean = 41.88 years old, *SD* = 11.87; 59 males; mean = 42.34, *SD* = 10.91) participated in this study. Sociodemographic and clinical characteristics of the sample can be consulted in Table 1. All the participants had previously received the OCD diagnosis by a qualified clinician following the OCD criteria established by the Diagnostic and Statistical Manual of Mental Disorders, fourth edition (DSM-IV), revised fourth edition (DSM-IV-TR) and fifth edition (DSM-5). All patients were previously assessed (at least 1 year before March 2020) by experienced clinicians in the OCD Clinical and Research Unit of the Department of Psychiatry, Hospital de Bellvitge, Barcelona, Spain, and were under pharmacological treatment and/or cognitive behavioral therapy. By the moment of the evaluation, patients who were under pharmacological treatment should be receiving a maintained dose for at least 3 months before the application of the semi-structured interview. Table 1 shows the type of treatment prescribed in the sample, the number of people with CBT associated with pharmacological treatment and the different comorbidities in the sample.

**TABLE 1:** Sociodemographic data of the sample.

Sociodemographics	*N* = 127
Sex	68 females 59 males
Age (y, m, SD)	42.0 ± 11.3
Years of education (y, m, SD)	12.7 ± 2.8
Age at OCD onset (y, m, SD)	17.5 ± 6.2
Working status	44 paid-employed 10 self-employed 4 students 16 unemployed 53 pensioners
Living status	17 alone 40 birth family 67 own family 3 with friends
Living with other people with psychiatric disorders	14 yes/113 no
Pre-pandemic Y-BOCS (m)	17.90 ± 6.2
Pre-pandemic HDRS (m)	10.83 ± 5.3
Pre-pandemic pharmacological treatment	53 SSRI 18 SRI 56 SRI/SSRI + antipsychotic
Pre-pandemic cognitive behavioral therapy	105
Pre-pandemic comorbidities	13 depression 9 dysthymia 3 bipolar disorder 6 anxiety disorder 1 phobia (simple) 1 social phobia 1 Post-traumatic stress disorder 4 Eating behavior disorder 3 Attention-deficit/hyperactivity disorder 2 Tics

*SSRI, Selective Serotonin Reuptake Inhibitors; SRI, Serotonin Reuptake Inhibitors.*

Exclusion criteria included dependence or history of substance abuse in the 6 months prior to the study and diagnosis of any psychotic disorders or autism spectrum disorders. Verbal consent for participation was formally recorded by research and clinical staff. Written consent was obtained in accordance with the Declaration of Helsinki, and the study was approved by the Ethics Committee of Hospital de Bellvitge.

### Questionnaires

As a consequence of the restrictions regarding face-to-face interviews, questionnaires and a semi-structured interview were administered by telephone, by a mental health professional known to the OCD patients. The questionnaire covered six thematic blocks focusing on the participant’s social and demographic data, social context during pandemic, contact with COVID-19, psychiatric condition prior to the pandemic, strategies used to regulate stress during quarantine and the clinical consequences on OCD symptomatology of the COVID-19 pandemic.

The variables forming part of the first five blocks were used as input variables to construct a predictive model for each output variable (consisting of symptoms caused by COVID-19). These variables will be referred to hereafter as the *full set of input variables*:

1. Sociodemographic data: age, gender, high-risk occupation and economic income during the pandemic.
2. Social context during the pandemic: cohabitants, relatives with mental illness, dependents, changes in family cohabitation and environment, perception of family support, and leaving home.
3. Contact with COVID-19: diagnosis of COVID-19 in a friend, relative or the participant themselves and daily time spent obtaining information about COVID-19 during the pandemic.
4. Previous psychiatric conditions: age of onset of OCD and duration in years, OCD subtype, current therapy and type of response, Y-BOCS and HDRS scores and daily hours dedicated to rituals.
5. Emotional regulation and coping strategies for stress: fears related to or caused by the COVID-19 pandemic and engagement in distracting activities.

### Machine Learning Techniques

Machine learning has been successfully applied to a wide variety of fields in the modeling of many real problems. A machine learning model learns to predict the output data as a function of the input data in a process called training, that uses a collection of examples that include input variables (in our case, participants’ information) and output variables (in our case, participants’ symptomatology or development of comorbidities). Once the model is trained, it can generalize its prediction to new data not used during training. When the output data takes a limited number of unordered values, they are considered as class labels and the models are called classifiers. When the output takes continuous values, the model is a regressor.

The classification was performed using the Linear Discriminant Analysis (LDA), implemented in the R statistical computing language, included as a simple linear classifier with a baseline performance. The Support Vector Machine (SVM) with radial basis function (RBF) kernel was also selected because it achieved a good performance in our previous generic comparative study (Fernández-Delgado et al., 2014). Specifically, the SVM was implemented by the LibSVM library (Chang and Lin, 2011) and accessed through its Octave binding, tuning the regularization parameter and the RBF kernel spread. The regression was performed using the multivariate Linear Regression Model (LM), also implemented in R, included as a linear method with baseline performance. The epsilon-Support Vector Regressor (SVR) was selected because it achieved a good performance in our recent comparison study (Fernández-Delgado et al., 2019). It also used RBF kernel, tuning the regularization parameter and RBF kernel width as in classification.

With the aim of anticipating the situation of patients diagnosed with OCD in a pandemic context, several output variables that are useful for clinicians were predicted:

1. Patients’ scores on the Y-BOCS during pandemic, that anticipate the changes in symptomatology. This variable takes continuous values ranging from 0 to 40 and gives a regression problem.
2. Appearance of obsessions and/or compulsions related to COVID-19, with values “yes” or “not,” that gives a classification problem.
3. Scores of self-perceived anxiety symptoms, taking continuous values between 0 and 10 and giving a regression problem.
4. Self-perceived depression symptoms, also taking continuous values between 0 and 10 and giving a regression problem.
5. Suicidal thoughts take three possible values “no suicidal thoughts,” “thoughts about death” or “thoughts about suicide,” that gives a classification problem.
6. Need for urgent psychiatric care, with values “yes” or “not,” that gives a classification problem.

Prediction of each of these outputs requires the construction of a specific machine learning classifier or regressor, depending on the output. Both models for classification (LDA and SVM) and regression (LR and SVR) use the whole set of variables including continuous and discrete ones.

### Evaluation Methodology

The performance of machine learning models in classification problems is measured using Cohen’s kappa value (*K*), that evaluates the agreement between the true and predicted categories labels excluding the agreement by chance (McHugh, 2012). The kappa (in %) is defined for classification problems as:


$$\kappa =100\,\frac{p_{a}-p_{e}}{s-p_{e}}\,p_{a}=\sum_{i=1}^{N}C_{i\,i}\,p_{e}=\frac{1}{N^{2}}\,\sum_{i=1}^{C}\left(\sum_{j=1}^{C}C_{i\,j}\right)\,\left(\sum_{j=1}^{C}C_{j\,i}\right)\,s=\sum_{i=1}^{C}\sum_{j=1}^{C}C_{i\,j}$$

where *C* is the number of categories, while *C*_*ij*_ is the amount of patients of class *i* that are predicted as of class *j*. The higher kappa, the better classifier, that is perfect when kappa = 100%. Classical definition for the kappa intervals and their significance (McHugh, 2012) considers that a kappa value between 1 and 20 means that the concordance between predicted and real category is slight; from 21 to 40, the concordance is fair; from 41 to 60, it is moderate; from 61 to 80, it is substantial; and more than 80 the classification is almost perfect. When the number of classes is *C* = 2 (sick and healthy), let the true positive (TP) be the number of sick people correctly identified as sick, the false positive (FP) be the number of healthy people incorrectly identified as sick, the true negative (TN) be the number of healthy people correctly identified as healthy, and false negative (FN) be the number of sick people incorrectly identified as healthy. The confusion matrix (Table 2) allows the visualization of performance of the classifier’s prediction:

**TABLE 2:** Confusion matrix for a two-class classification problem, where the number in the *i*-th row and *j*-th column is *C*_*ij*_.

		Predicted class	
		Sick	Healthy
**True class**	Sick	TP	FN
	Healthy	FP	TN

Each column in the matrix represents the patients in the predicted class while each row represents the patients in the true class. The sensitivity (Se) refers to the classifier’s ability to correctly detect sick patients while the specificity (Sp) relates the classifier’s ability to correctly reject healthy patients, according to these definitions:


$$S\,e=\frac{100\,T\,P}{T\,P+F\,N}\,S\,p=\frac{100\,T\,N}{T\,N+F\,P}$$

Finally, another performance measure for classification with two-classes is the area under the ROC curve (AUC), also in %. The ROC curve is defined by the sensitivity plotted against 100-specificity when the classifier behavior changes from assigning all the patterns to one class to assign all the patterns to the other class. In regression problems, the most popular measures are the Fisher correlation coefficient (R) and the root mean squared error (*RMSE*), defined as:


$$R=\frac{\sum_{i=1}^{N}\left(y_{i}-\bar{y}\right)\,\left(o_{i}-\bar{o}\right)}{\sqrt{\left[\sum_{i=1}^{N}{\left(y_{i}-\bar{y}\right)}^{2}\right]\,\left[\sum_{i=1}^{N}{\left(o_{i}-\bar{o}\right)}^{2}\right]}}$$

$$R\,M\,S\,E=\sqrt{\frac{1}{N}\,\sum_{i=1}^{N}{\left(y_{i}-o_{i}\right)}^{2}}$$

where *N* is the number of patients, *y*_*i*_ and *o*_*i*_ are, respectively, the predicted value by the model and the true value for *i*-th patient, while $\bar{y}$ and $\bar{o}$ are the average values of *y*_*i*_ and *o*_*i*_, respectively. Classical definition (Colton, 1974) for the correlation intervals and their significance considers that a R value between 0 and 0.15 means that the true and predicted values for each patient are not correlated at all; R between 0.15 and 0.5 means bad to moderate correlation between them; R between 0.5 and 0.75 means moderate to good correlation; and R > 0.75 means very good to excellent correlation. Another measure of the prediction accuracy is MAE (Mean Absolute Error), defined as the average absolute difference between the predicted and true output values:


$$M\,A\,E=\frac{1}{N}\,\sum_{i=1}^{N}\left|y_{i}-o_{i}\right|$$

In our study, we use a fourfold cross-validation to estimate the model performance. In the *i*-th trial, with *i* = 1,…,4, the *i*-th fold is left for testing, and the remaining threefold are used to configure (i.e., to train and tune) the model. Since most of the models have one or several hyper-parameters whose values must be tuned in order to get the best available performance, two of these threefold are used to train the model using a given combination of hyper-parameter values, while the remaining fold is used as the so called “validation set” to evaluate the performance of this trained model. The process is repeated for the different combinations of hyper-parameter values, and the combination with the highest performance on the validation set is selected. Finally, the model trained with the training and validation set using the best combination of hyper-parameter values is tested on the *i*-th fold, which was devoted to the test. This process is repeated for *i* = 1,…,4 and the test performance is averaged over the fourfold.

## Results

### Sociodemographic and Clinical Characteristics

The sample used in this study comprised 127 participants (53.5% women). The participants ranged in age from 18 to 64 years (average age 42 years, *SD* = 11.3 years). The mean age of onset of the disorder was at age 17.5 years (*SD* = 6.2). The mean severity of OCD prior to the pandemic was 17.9 points, and the mean score for symptoms of depression was 10.83 points. Of the 127 participants, 17 lived alone (13%), 40 lived with their birth family (32%), 67 lived with their own family (53%), and 3 lived with friends (2%). In addition, 54 participants were employed (42%), 16 were unemployed (13%), 53 were pensioners (42%), and 4 were students (3%). Finally, 14 participants (11%) were living with other people with psychiatric disorders.

### Prediction of Symptoms Based on Regression Analysis

**FIGURE 1:**
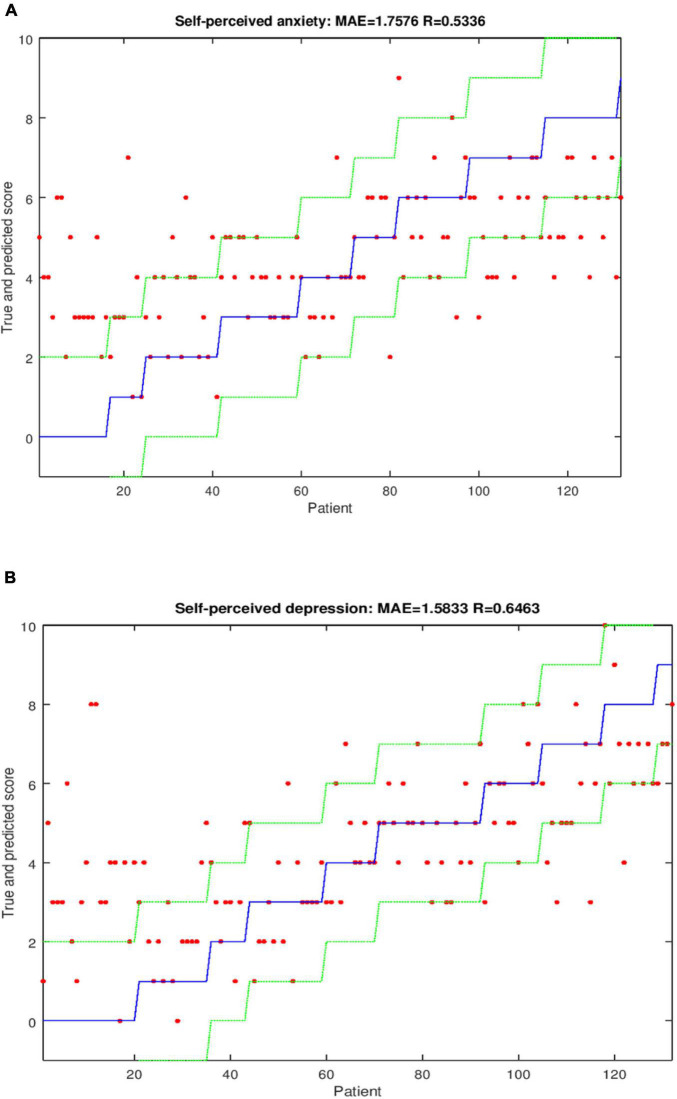
**(A)** Graphical representation of *Y-BOCS during the pandemic* without inclusion of gender and using the regressor SVR. **(B)** Percentage of patients correctly classified within different tolerances for *Y-BOCS during pandemic* using the SVR regressor.

Regarding the regression analysis, the correlation (R) and Mean Absolute Error (MAE) for the outputs *Y-BOCS during the pandemic*, *self-perceived depression* and *self-perceived anxiety* are shown in Table 3. Analyses were conducted with and without gender as an input variable (columns with and without G in the tables below, respectively). Prediction of the *Y-BOCS during the pandemic* was more reliable when the *SVR* regressor was used, without any clear importance in relation to gender (*R* = 0.94 in both cases, MAE = 2.13 including gender and MAE = 2.14 excluding this information). Following the Colton criterion (Colton, 1974), the prediction varied from good to excellent. In order to represent the meaning of this data from a clinical perspective, the reliability of the prediction reported by the model is shown in Figures 1A,B. The output *Y-BOCS during the pandemic* is represented in Figure 1A. The blue line represents the true values, the green lines define a tolerance threshold of ± 3, and red dots are the values of *Y-BOCS during the pandemic* predicted by the model. The red dots lying within green lines represent patients for whom the prediction of *Y-BOCS during the pandemic* was wrong in less than 3 of the true scores (indicating that 76.47% of the patients were correctly classified within this tolerance threshold). This tolerance threshold is related to MAE. Correct classifications for different tolerances (*t*) were as follows: 15.44% of the patients were classified within *t* = 0, where true and predicted values were equal. In the case of *t* = 1 (*Y-BOCS during the pandemic* was predicted within a tolerance threshold of ± 1), 44.85% of the patients fit within this threshold, while 65.44% of the patients were correctly classified with *t* = 2, and 76.47% of the patients were correctly classified within *t* = 3. The percentage of patients correctly classified within different tolerances in *Y-BOCS during pandemic* is represented in Figure 1B. Almost 100% of the patients were correctly classified within a tolerance threshold of ± 6.

**TABLE 3:** Correlation R and MAE using the SVR and LR regressors with and without gender (columns I and I+G, respectively).

		R		MAE	
Output	Regressor	I	I + G	I	I + G
Y-BOCS during pandemic	SVR	**0.94**	**0.94**	2.14	**2.13**
	LR	0.58	0.54	5.29	5.88
Self-perceived depression	SVR	0.65	**0.68**	1.58	**1.51**
	LR	0.46	0.37	2.11	2.15
Self-perceived anxiety	SVR	0.53	**0.58**	**1.76**	1.85
	LR	0.36	0.42	2.17	2.03

*The best results (highest R and lowest MAE) are shown in bold.*

*I, inputs; G, gender.*

**FIGURE 2:**
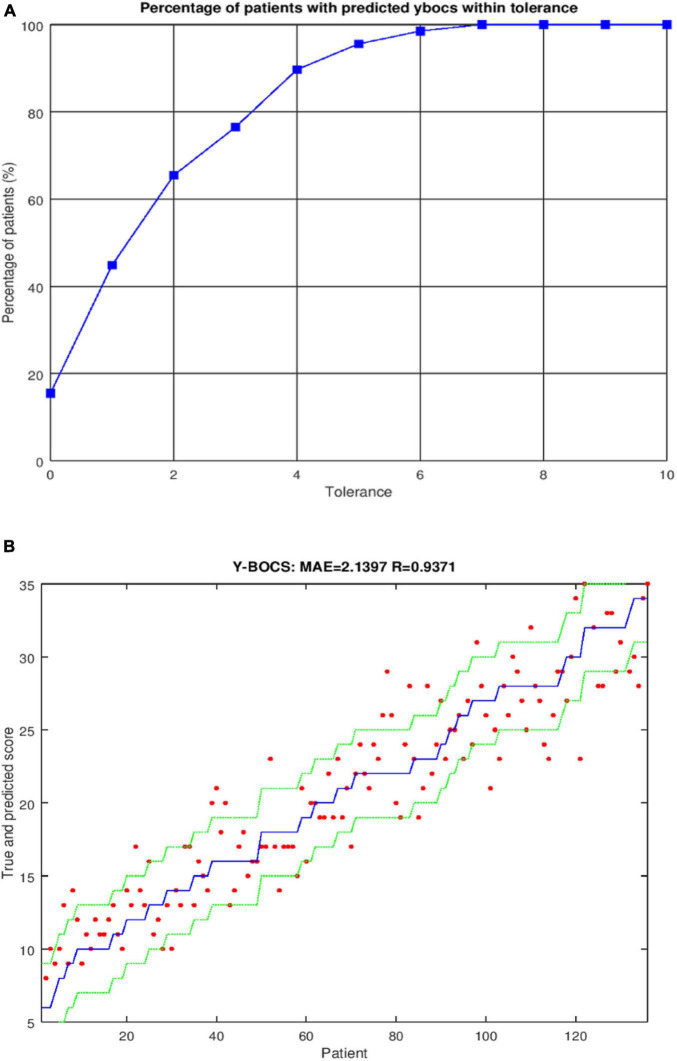
**(A)** Graphical representation of *self-perceived depression* using the regressor SVR. Correct classifications were obtained for different tolerances: for *t* = 0, 26.52% of the patients were correctly classified; for *t* = 1, 57.58% were correctly classified; for *t* = 2, 80.30% were correctly classified; and for *t* = 3, 90.91% were correctly classified. **(B)** Graphical representation of *self-perceived* anxiety using the regressor SVR. Correct classifications were obtained for different tolerances: for *t* = 0, 21.97% of the patients were correctly classified; for *t* = 1, 43.94% were correctly classified; for *t* = 2, 70.45% were correctly classified; and for *t* = 3, 87.12% were correctly classified.

For *self-perceived depression*, the prediction was improved by using the *SVR* regressor and including gender (MAE = 1.51 and *R* = 0.68). Prediction of *self-perceived anxiety* was improved by using the *SVR* regressor but only considering the inputs taking MAE as criterion (MAE = 1.76 and *R* = 0.53). When R was used as a criterion, the prediction was improved by including gender (MAE = 1.85 and *R* = 0.58). Following the Colton criterion, the prediction varied from moderate to good for *self-perceived depression* and *self-perceived anxiety*. All of the results for prediction of each variable are presented in Table 3. Graphical representations for predicting these two outputs are shown in Figures 2A,B (Figure 2A for *self-perceived depression* and Figure 2B for *self-perceived anxiety*) with four different thresholds: *t* = 0, *t* = 1, *t* = 2, and *t* = 3.

### Prediction of Symptoms Based on Classification Analysis

The kappa value obtained when SVM and LDA were used for the classification of *new obsessions and/or compulsions related to COVID-19 infection* (2 classes), *suicidal thoughts* (3

classes) and *need for urgent psychiatric care* (2 classes), and are reported in Table 4. In the first and third datasets, that have 2 classes, the AUC is also reported by Table 4. Prediction of *obsessions and/or compulsions related to COVID-19* was perfect (kappa = AUC = 100%) with the SVM classifier including inputs and gender. For the remaining outputs, the results were adequate, with kappa values of between 21 and 40%: *suicidal thoughts* (kappa = 38.9% using *LDA* without gender information), and *need for urgent psychiatric care* (kappa = 32.3% using SVM including gender information). In this case, both classes are very unbalanced (only 6.3% of patients required urgent care). Thus, the AUC (80.9%), that does not consider the class unbalance, is much higher than kappa.

**TABLE 4:** Kappa and AUC (only for two-class problems) values, in percentages, yielded by SVM and LDA classifiers with and without gender as input (columns I and I+G, respectively).

		Kappa		AUC	
Output	Classifier	I	I + G	I	I + G
Obsessions and/or compulsions related to COVID-19	SVM	98.4	**100**	99.8	100
	LDA	93.4	81.9	97.6	93.2
Suicidal thoughts	SVM	34.2	38.7		
	LDA	**38.9**	30.8		
Need for urgent psychiatric care	SVM	23.4	**32.3**	80.5	89.6
	LDA	17.9	17.1	64.9	60.8

*The best kappa values are shown in bold.*

*I, inputs; G, gender.*

With the aim of explaining the meaning of a specific kappa value, we present the confusion matrix for *suicidal thoughts* with the inputs and the classifier *LDA* (kappa = 38.9%) in Table 5. The values in the table are means for all patients. The sum of the elements in the diagonal represents the patients correctly classified by the computer (64.7% of patients), and the elements outside of the diagonal represent erroneously classified patients (35.3%). Type 3 patients (suicidal thoughts) were classified as type 2 (thoughts about death) or type 3, but never as type 1 (no suicidal thoughts). Only 11.8% of the patients were erroneously classified by the computer as type 2 or 3, after not reporting any thoughts about death or suicidal thoughts, threat or attempts. Thus, the classifier yielded a sensitivity of 78.9% and a specificity of 88.1% in predicting the absence of suicidal thoughts.

**TABLE 5:** Confusion matrix for *Suicidal thoughts* using the LDA classifier and excluding gender from the inputs (kappa = 38.9%).

		Predicted category			Sensitivity	Specificity
		Type 1	Type 2	Type 3		
True category	Type 1	44.1	7.4	4.4	78.9	88.1
	Type 2	10.3	16.2	8.8	45.9	57.8
	Type 3	0.0	4.4	4.4	50.0	25.0

*Type 1, no suicidal thoughts; Type 2, thoughts about death; Type 3, suicidal thoughts, threats or attempts.*

### Re-analysis Using the Most Relevant Variables in Each Output

The objective of the analysis was to make predictions based on a large number of variables covering all the questions in the questionnaire, referred to in this study as the full set of input variables. In addition, we analyzed which variables had the greatest weight in predicting each of the outputs, in order to create reliable models with as few variables as possible. To calculate the relevance of the variables, we used the *fss* function of the *nan* package of the Octave software, that uses the Feature Subset Selection and Feature Ranking method (Peng et al., 2005) based on Pearson’s correlation. For each output, the best classifier or regressor was tested removing the variables sorted by increasing relevance (i.e., less relevant variables are removed first).

**FIGURE 3:**
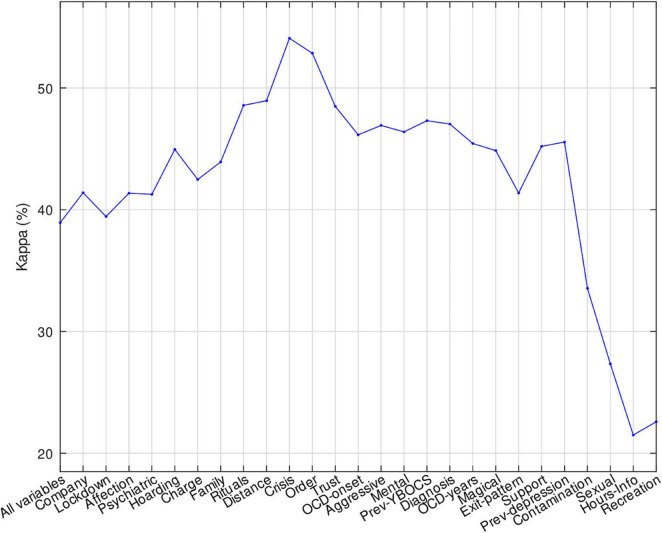
Performance of LDA to predict *Suicidal thoughts* reducing the set of variables by increasing relevance.

The expectable behavior is that performance gradually degrades as the number of variables reduces. This degradation should be slow at the beginning, where the less relevant variables are discarded, and fast when more relevant variables are removed. This behavior was observed in the majority of the outputs, such as *Perceived depression* or *Need for urgent psychiatric care*. There were, however, some exceptions. Regarding classification, the performance to predict *Suicidal thoughts* (Figure 3) raised from 38.9 to 54.1% by keeping the 16 most relevant variables (*Trust, OCD onset, Aggressive, Mental, Previous YBOCS, Diagnosis, OCD years, Magical, Exit pattern, Support, Previous depression, Contamination, Sexual, Hours info, Recreation*, and *Fear*). In Supplementary Material it can be seen the complete variables to which each keyword refers and the type of variable (i.e. binary, continuous or ordinal) each one is. As well, using only the most relevant variable (*Sexual Subtype*), the SVM predicted *Obsessions and compulsions* with the same kappa (100%) as using the whole set (28 variables). Considering regression, the SVR using only the most relevant variable *Ritual hours* achieved *R* = 0.90, very near to the maximum value *R* = 0.94 achieved with all the variables, in the prediction of *Y-BOCS during the pandemic*, even excluding the *Previous Y-BOCS* variable.

## Discussion

This validity study aimed to demonstrate the reliability and usefulness of using machine learning models to predict symptomatology changes in a cohort of OCD patients during the COVID-19 pandemic based on pre-pandemic sociodemographic and clinical data. Worsening of OCD symptoms prediction or the emergence of new symptomatology is important in situations such as pandemics, as it could help clinicians to prescribe individually tailored interventions to patients at risk. The results of our predictive analysis based on the full set of input variables was useful for a reliable prediction of the outputs. Specifically, the interview provided very good reliability regarding prediction of changes in general OCD symptomatology and in COVID-19 related symptoms, as well as prediction of OCD comorbid symptoms such as depression or anxiety.

Regarding the reliability prediction of *Y-BOCS during the pandemic*, almost 100% correct classification was achieved in a ± 6 threshold. The model may fail to classify the patient’s severity range especially if the score difference is very small. However, if the difference falls above or below 6, the model shows very good prediction, wish can be crucial to detect changes in the severity level of OCD, especially in the cases in which subjects have scores in the YBOCS that are in the cutting edge to change to a different severity level (e.g., change from mild to moderate or moderate to severe).

Prediction of changes in symptoms during the initial stages of the pandemic was important to enable provision of appropriate and timely support to high-risk patients. Evidence of worsening of OCD symptoms during the pandemic has been reported, especially in symptoms, such as compulsive hand-washing, related to the measures implemented to prevent COVID-19 infections (French and Lyne, 2020; Alonso et al., 2021; Wheaton et al., 2021), in both adult and pediatric populations (Nissen et al., 2020; Sulaimani and Bagadood, 2020). In addition, recovered and partially recovered patients experienced significant worsening of symptoms during the pandemic (Matsunaga et al., 2020; Tanir et al., 2020) such patients should be included in the population at risk of worsening symptomatology. Previous studies have systematically reported that stress can worsen OCD symptoms and, also, that stressful situations may act as a trigger for the onset of (new) OCD symptoms (Davide et al., 2020). This is particularly important in a context such as the early stages of a pandemic.

Anxiety and depression were by far the most commonly reported psychological symptoms in OCD patients (Silva et al., 2020; Alonso et al., 2021) during the COVID-19 pandemic. As OCD patients are more vulnerable to developing emotional problems throughout their life, it is anticipated that they may be more prone to experiencing emotional and mood disturbances during exposure to prolonged stressful situations (Silva et al., 2020), such as a pandemic. With our methodology, we can also fairly reliably predict how patients with OCD will perceive their levels of anxiety and depression. If this self-perception is elevated, the patients are likely to need more psychiatric support. In the case of the self-perception of anxious symptoms, as the chance of wrong prediction is not very high, we may still detect alarm signs that require clinical evaluation. Regarding the self-perception of depressive symptoms, as the range of error was also low when the gender of the patient was included, we can consider that the prediction of these emotional symptoms was quite reliable. Rossi et al. (2020) reported an increase in depressive and anxious symptoms in the Italian population with a positive association with gender (with females at greater risk of suffering emotional comorbidities).

Regarding the remaining outputs (*need for urgent psychiatric care* and *suicidal thoughts*), the models yielded a low level of agreement between the true and the predicted value. For *need for urgent psychiatric care*, the reliability was improved by including gender. Predicting comorbid symptomatology is very important for the prognosis of OCD patients. Recent studies have reported sleep problems (Banerjee, 2020; Alonso et al., 2021), suicidal ideation (Alonso et al., 2021) and emotional symptoms (Silva et al., 2020; Alonso et al., 2021), as the symptoms emerging during the COVID-19 pandemic in OCD patients. In the present study, the values predicted for some symptoms (e.g., suicidal ideation) were weakly correlated with the true values. For those variables for which the model did not yield highly reliable predictions, professionals must be alert to the symptoms that may arise in a future situation of confinement. Considering the capacity of the model to classify the three possible groups of suicidal thoughts (i.e., absence of suicidal thoughts, thoughts about death or suicidal ideation), the model fairly highly reliably predicted which patients will not present suicidal ideation. However, the model was less reliable for differentiating between patients who have thoughts about death and those who present autolytic ideation. Mental health professionals should be aware of the need to identify the type of thoughts that patients may present.

Gender information seems to be irrelevant for predicting many outputs, as the differences in model quality (kappa in classification and correlation in regression) were generally very small. Thus, gender information may be implicitly included in some of the other input variables, providing the information needed in those cases.

This study has the following limitations: generality of the results to the OCD population needs to be considerate with caution, and the since the sample size used in this preliminary study is small. In addition, due to pandemic-related restrictions, the evaluations were performed by phone, which may have been less effective that face-to-face clinical evaluation in detecting clinical changes in the symptomatology. Also, we did not include the type of pharmacological treatment in the model, nor the comorbid disorders that patients presented. We did not consider variations in pharmacological treatment (type and dosage) over the examined period due to patients’ worsening that could be interesting to predict.

### Future Implications

We constructed relatively reliable and accurate predictive models for OCD symptoms during the COVID-19 pandemic, providing useful information about the most relevant variables for predicting worsening of symptomatology among OCD patients during a pandemic situation. The present results should be taken into account in developing preventive measures for the OCD population at risk of worsening symptoms. Prediction of other symptoms such as emotional traits could prevent suffering in OCD patients, who would otherwise have to seek urgent care in mental health units in a pandemic situation. Prediction of symptoms during a pandemic may not require an extensive battery of clinical measures in order to distinguish between patients at higher risk of worsening of symptoms from those at lower risk and, therefore, to ensure provision of timely and appropriate clinical support.

Prediction of symptoms also has important benefits to the general population. The maintenance of scheduled consultations by telephone as opposed to unnecessary constant monitoring of OCD patients could help decongest mental health services, enabling more efficient distribution of human resources to attend to people who develop new comorbidities and new mental health problems caused by the pandemic (Moreno et al., 2020), such as pandemic fatigue. Prior knowledge of OCD patients at risk will enable more targeted control even without the need for face-to-face consultation. This will help prevent possible infections by minimizing exposure in the hospital environment and also help to stabilize OCD symptoms by allowing patients to remain in the controlled home environment.

Machine learning models are important techniques for identifying sets of predictors in OCD patients, with implications for clinicians and researchers. Our study may serve as proof of viability for future applications of the machine learning models developed. This method could be used to develop a software application that could be used in psychiatric services, acting as an alarm system to predict changes or new symptomatology to be developed by patients under a future pandemic situation, and clinicians should be aware of this information.

## Conclusion

In conclusion, our study provides evidence that artificial intelligence can be used efficiently to predict the psychological impact of a pandemic in OCD patients. Some of the most important symptoms in this context can be predicted by using a machine learning model in changes in Y-BOCS scores and the appearance of OCD symptomatology related to COVID-19. These models may be of great support for clinicians to provide optimized and personalized care to people with a high risk of suffering mental health problems during situations such as a pandemic. When prediction of symptoms is not reliable, clinicians should consider other symptoms that patients may display. Further validation of the models is required in order to enable clinical implementation of the current findings.

## Data Availability Statement

The raw data supporting the conclusions of this article will be made available by the authors, without undue reservation.

## Ethics Statement

The studies involving human participants were reviewed and approved by the Ethics Committee of Hospital de Bellvitge. The patients/participants provided their written informed consent to participate in this study.

## Author Contributions

MT-F: conceptualization, methodology, investigation, writing—original draft, writing—review and editing, and visualization. EC: conceptualization, methodology, software, validation, formal analysis, writing—original draft, and writing—review and editing. ÓG: resources, writing—original draft, writing—review and editing, and supervision. CS, SB, LM-B, and ER: investigation, resources, and writing—review and editing. MF-D: methodology, software, validation, formal analysis, and writing—review and editing. JM and AC: resources, writing—review and editing, and supervision. SC: conceptualization, methodology, resources, writing—original draft, and writing—review and editing. PA: conceptualization, methodology, investigation, resources, writing—original draft, and writing—review and editing. MF-P: conceptualization, methodology, investigation, writing—original draft, writing—review and editing, visualization, and project administration. All authors contributed to the article and approved the submitted version.

## Conflict of Interest

The authors declare that the research was conducted in the absence of any commercial or financial relationships that could be construed as a potential conflict of interest.

## Publisher’s Note

All claims expressed in this article are solely those of the authors and do not necessarily represent those of their affiliated organizations, or those of the publisher, the editors and the reviewers. Any product that may be evaluated in this article, or claim that may be made by its manufacturer, is not guaranteed or endorsed by the publisher.
