# Supplementary material for: Viability Study of Machine Learning-Based Prediction of COVID-19 Pandemic Impact in Obsessive-Compulsive Disorder Patients
Source: Front Neuroinform. 2022 Feb 10;16:807584. doi: 10.3389/fninf.2022.807584 (PMC8866769; doi:10.3389/fninf.2022.807584)
Supplement: Supplementary file 1 [file Table_1.DOCX]

Supplementary material

| **INPUTS** | **CLUE** | **Type of variable** |
| --- | --- | --- |
| Do you have a relative with a mental disorder? | Mental | Binary |
| Do you have people in your charge? | Charge | Binary |
| Do you have people you trust to speak to? | Trust | Binary |
| Do you have company? | Company | Binary |
| Do you feel supported by your family members? | Support | Binary |
| Personal, family or friend with a COVID-19 diagnosis | Diagnosis | Binary |
| Exit pattern during lockdown | Exit | Ordinal |
| OCD onset age | OCD-onset | Continuous |
| Years of OCD duration | OCD-years | Continuous |
| No. of daily hours dedicated to being informed about COVID-19 | Hours | Continuous |
| Affection of coexistence | Affection | Binary |
| Previous Y-BOCS | Previous-Y-BOCS | Continuous |
| Contamination/Washing OCD Subtype | Contamination | Ordinal |
| Hoarding OCD Subtype | Hoarding | Ordinal |
| Aggressive thoughts/checking OCD Subtype | Aggressive | Ordinal |
| Magical thinking and repetition/order/symmetry rituals OCD Subtype | Magical | Ordinal |
| Order without magical thinking OCD Subtype | Order | Ordinal |
| Sexual/religious OCD Subtype | Sexual | Ordinal |
| No. hours per day dedicated to rituals | Rituals | Continuous |
| Fear of getting COVID-19 | Fear | Ordinal |
| Fear of a family member getting COVID-19 | Family | Ordinal |
| Fear of lockdown and loss of routines | Lockdown | Ordinal |
| Fear of difficulty getting psychiatric care as usual | Psychiatric-care | Ordinal |
| Affection by forced family distance | Distance | Ordinal |
| Fear of an economic crisis caused by COVID-19 | Crisis | Ordinal |
| Recreation activities | Recreation | Binary |
| Previous depression | Depression | Continuous |
